# No inbreeding depression in laboratory‐reared individuals of the parasitoid wasp *Allotropa burrelli*

**DOI:** 10.1002/ece3.2643

**Published:** 2017-01-15

**Authors:** Bastien Quaglietti, Lucie Tamisier, Géraldine Groussier, Alexandre Fleisch, Isabelle Le Goff, Nicolas Ris, Philippe Kreiter, Xavier Fauvergue, Thibaut Malausa

**Affiliations:** ^1^ UMR 1355‐7254 Institut Sophia AgrobiotechCNRSINRAUniversité Nice Sophia AntipolisSophia AntipolisFrance

**Keywords:** *Allotropa burrelli*, biological control, captive populations, genetic load, haplodiploidy, inbreeding depression

## Abstract

Inbreeding depression is a major concern in almost all human activities relating to plant and animal breeding. The biological control of pests with natural enemies is no exception, because populations of biocontrol agents experience a series of bottlenecks during importation, rearing, and introduction. A classical biological control program for the Comstock mealybug *Pseudococcus comstocki* (Hemiptera: Pseudococcidae) was initiated in France in 2008, based on the introduction of an exotic parasitoid, *Allotropa burrelli* Mues. (Hymenoptera: Platygastridae), a haplodiploid parasitoid imported from Japan. We evaluated the sensitivity of *A. burrelli* to inbreeding, to optimize rearing and release strategies. We compared several morphological and life‐history traits between the offspring of siblings and the offspring of unrelated parents. We took into account the low level of genetic variability due to the relatively small size of laboratory‐reared populations by contrasting two types of pedigree: one for individuals from a strain founded from a single field population, and the other generated by hybridizing individuals from two strains founded from two highly differentiated populations. Despite this careful design, we obtained no evidence for a negative impact of inbreeding on laboratory‐reared *A. burrelli*. We discussed the results in light of haplodiploid sex determination and parasitoid mating systems, and classical biological control practices.

## 1 Introduction

Bottlenecked populations, whether natural or artificially reared, may display panmictic inbreeding, when genetically related individuals mate despite the occurrence of random mating (Malécot, 1969). Severe consequences may occur such as inbreeding depression, defined as a lower fitness of inbred than of outbred individuals (Charlesworth & Charlesworth, 1987; Keller & Waller, 2002), which itself increases the risk of population extinction (Bijlsma, Bundgaard, & Boerema, 2000; Fauvergue, Vercken, Malausa, & Hufbauer, 2012; Frankham, 2005; Nieminen, Singer, Fortelius, Schöps, & Hanski, 2001; Wright, Tregenza, & Hosken, 2007).

Inbreeding depression arises when the increase in homozygosity in inbred individuals leads to at least one of the following mechanisms: (1) the expression of detrimental recessive alleles (partial dominance) or (2) a general advantage for heterozygosity which translates into lower relative fitness for highly homozygous inbred individuals (overdominance; Charlesworth & Willis, 2009). As partial dominance is the most pervasive mechanism (Charlesworth & Charlesworth, 1987; Crnokrak & Roff, 1999), the intensity of inbreeding depression thus depends on the population “genetic load.” It is defined as the frequency of detrimental recessive alleles in the genetic pool (Charlesworth & Charlesworth, 1987; Charlesworth & Willis, 2009; Glémin, 2003) which is mainly influenced by purge, that is, the elimination of detrimental alleles by drift or by selection (Crnokrak & Barrett, 2002; Glémin, 2003; Swindell & Bouzat, 2006). The strength of purge is influenced by many factors, including the effect size of detrimental alleles, their degree of recessiveness, and population size.

Population size has a complex effect on the purging of detrimental alleles. Inbreeding depression is most likely to occur in small populations, which have higher levels of genetic relatedness, and in recently bottlenecked populations in which genetic drift may overcome selection and favor an increase in the frequency of deleterious mutations, or even their fixation (Fauvergue et al., 2012; Luque et al., 2016). By contrast, a rapid increase in population size after a bottleneck may purge the genetic load, leading to a decrease in inbreeding depression (Facon et al., 2011; Kirkpatrick & Jarne, 2000; Pujol, Zhou, Sanchez, & Pannell, 2009). Thus, inbreeding depression in small populations may be either stronger or weaker than that in populations at demographic equilibrium, depending on the magnitude and duration of the bottlenecks experienced.

Ploidy (diploid vs. haplodiploid) may also affect the purging process. Haplodiploid organisms should be less sensitive to inbreeding, because recessive deleterious alleles are expressed in haploid males and purged through selection (Crozier, 1985; Werren, 1993). However, theoretical models suggest that haplodiploids should nevertheless suffer from inbreeding depression, because the proportion of deleterious alleles is decreased, but these alleles are not completely eliminated (Glémin, 2003; Werren, 1993). In addition, genes involved in female‐specific traits would not be purged through haploid males, so inbreeding depression is more likely for such traits (Werren, 1993). Secondly, in many gregarious or quasi‐gregarious species (which deposit more than one egg per host or per host patch), males mate locally with their sisters emerging from the same host individual or host patch. This behavior induces regimes of high inbreeding, which could accelerate purging. Gregarious organisms would therefore be expected to display lower levels of inbreeding depression than solitary species.

Henter (2003) performed a meta‐analysis to test these predictions in insects, by comparing inbreeding depression between diploid and haplodiploid species and between solitary and gregarious species. As expected, diploids were more strongly affected by inbreeding than haplodiploids, although significant inbreeding depression was observed in some haplodiploids (Gerloff & Schmid‐hempel, 2005; Greeff, Jansen van Vuuren, Kryger, & Moore, 2009; Henter, 2003). Nevertheless, the authors found no difference between solitary and gregarious species. Finally, a difference in sensitivity to inbreeding between female and male traits was reported in one study, on the mite *Tetranychus urticae*, in which inbreeding affected oviposition rate but not traits common to males and females (Tien, Sabelis, & Egas, 2014).

Inbreeding depression has been studied in the context of livestock breeding (Croquet, Mayeres, Gillon, Vanderick, & Gengler, 2006; Dorostkar et al., 2012), plant breeding (Basamma, Kajjidoni, Salimath, & Malagouda, 2009; Jain & Bharadwaj, 2014), conservation biology (Hedrick & Kalinowski, 2000), invasive species management (Facon et al., 2011), and biological control (Facon et al., 2011; Fauvergue et al., 2012; Henter, 2003). In biological control, inbreeding depression may be an issue during laboratory and industrial rearing (de Clercq, Vandewalle, & Tirry, 1998) as well as during field releases (Fauvergue & Hopper, 2009), leading to a decrease in biocontrol performance and consequent chance of extirpation of the control species. Although concerns have frequently been raised about inbreeding depression in biological control (Hopper, Roush, & Powell, 1993; Mackauer, 1976), experimental data are scarce and diverse patterns have been observed. For example, inbreeding was found to have no effect on phenotypic traits in the predator *Podisus maculiventris* (Say; Heteroptera: Pentatomidae) during the monitoring of inbred lines and mass‐reared populations for 30 generations (de Clercq et al., 1998). By contrast, decreases in adult emergence and host mortality were observed after two generations of inbreeding in the fly *Exorista japonica* (Nakamura, 1996). Inbred lines even went extinct after six generations of inbreeding, whereas conventionally bred lines continued to thrive. Similarly, a laboratory study on *Cryptolaemus montrouzieri* (Coleoptera: Coccinellidae) reported the rapid development of differences in fecundity, longevity, and predation capacities between sib‐mating lines and lines displaying random allogamy (Al‐Khateeb, Asslan, El‐Heneidy, & Basheer, 2013).

We investigated the effect of inbreeding in the parasitoid wasp *Allotropa burrelli* Muesebeck (Hymenoptera: Platygastridae). As a specialist natural enemy of the Comstock mealybug *Pseudococcus comstocki* (Hemiptera: Pseudococcidae), it was used as a biological control agent. *A. burrelli* originates to Northeast Asia and has been introduced into Central Asia (DeBach & Rosen, 1991) and North America (Meyerdirk & Newell, 1979), for the biological control of *P. comstocki*, and is currently used in a biological control program in France.

*Allotropa burrelli* has several features likely to render it insensitive to inbreeding depression. It is a gregarious endoparasitoid, and the females lay several eggs per host. The males generally emerge before the females and stay on the host, waiting for the females to emerge (Clancy, 1944). As in other species of the order Hymenoptera, the males hatch from unfertilized eggs and are haploid. This combination of systematic inbreeding and haplodiploidy should have facilitated the purging of deleterious alleles over the species' evolutionary history (Crnokrak & Barrett, 2002; Glémin, 2003; Malécot, 1969). However, this general expectation may be modified by the demography of *A. burrelli*: Strong bottlenecks resulting from host–parasitoid dynamics may cause genetic drift that may either eliminate or fix deleterious alleles (Boakes, Wang, & Amos, 2007; Kirkpatrick & Jarne, 2000). Furthermore, as mentioned above, haplodiploidy does not preclude inbreeding depression for female traits.

We thus evaluated the sensitivity of *A. burrelli* to inbreeding depression, by comparing fitness‐related traits between inbred (crosses between siblings) and outbred (random mating) individuals. This study confirms the predicted absence of inbreeding depression for this species and highlights an interesting way to overcome a problem that often limits inferences in similar studies. Inbreeding depression may go undetected in laboratory‐reared populations, because the deleterious alleles have been either eliminated or been fixed through genetic drift. We thus used two different populations and an ad hoc crossing design to detect putative deleterious alleles: The individuals tested (G_2_) were descended from siblings or from unrelated parents (G_1_), the parents of which (G_0_) originated either from the same strain or from two different strains (the G_1_ individuals were therefore either “pure” or “hybrid”). If the two strains used had lost or fixed different detrimental alleles at different loci, then inbreeding depression would be expected to be weak or absent in the progeny of pure parents. Inbreeding depression might nevertheless be detected in the progeny of hybrid parents, because these alleles would occur in the heterozygous state more frequently in outbred than in inbred progeny. The absence of inbreeding depression in the two crossing schemes made it possible to draw robust conclusions about the frequency of strong‐effect deleterious alleles in the gene pool.

## 2 Materials and Methods

### 2.1 Biological material

We used *P. comstocki* individuals sampled from a mass rearing founded with individuals collected in the field in southern France in 2008. We reared *P. comstocki* on sprouted potatoes in controlled conditions (25°C, 60% ± 10% R.H. and total darkness). Two laboratory strains of *A. burrelli* were founded in 2010 from natural populations sampled in Japan. We collected seven hundred individuals from the Utsunomiya District and 99 individuals from the Tottori District. The sex of specimens can be determined on the basis of a specific dimorphism of the antennae. Males have long, hirsute, and moniliform antennae, whereas females have shorter, sparsely pubescent, and distinctly club‐shaped antennae. Development lasts 30 days at 28°C. The two strains (“Utsunomiya” and “Tottori”) were mass‐reared in plastic boxes containing second‐ and third‐instar larvae of *P*. *comstocki*. We smeared honey on the box lids as a food source for the parasitoids. We carried out rearing at 24 ± 3°C, 70% ± 10% R.H., and 12‐hr light/12‐hr dark cycle.

### 2.2 Experimental design

**Figure 1:**
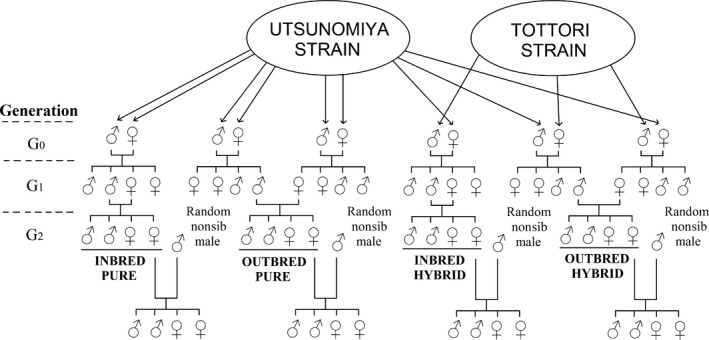
Crossing design. We established the families by crossing two G_0_ individuals from Utsunomiya (“pure families”), or from Utsunomiya and Tottori (“hybrid families”). At generation G_1_, we performed sib crosses (inbred crosses) and nonsib crosses (outbred crosses). Morphological and life‐history traits were measured on G_2_ individuals

We measured the impact of inbreeding on *A. burrelli*, by carrying out crosses between individuals from the two strains (Figure 1). We first established families from males and females sampled from the two strains (generation G_0_), crossed G_1_ individuals within and between families (sibling vs. outbred crosses), and measured phenotypic traits in the progeny (G_2_). Environmental stresses, such as extreme temperature, are known to increase sensitivity to inbreeding (Kristensen, Barker, Pedersen, & Loeschcke, 2008). Therefore, we chose to increase temperature and duration of the light period relatively to the rearing conditions in order to make experiment conditions more extreme. The experiment was carried out at 28 ± 3°C, 70% ± 10% R.H., and with a 14‐hr light/10‐hr dark cycle.

#### 2.2.1 Establishment of families

At generation G_0_, we set up two types of families, using individuals collected from mass‐reared strains: (1) “pure families,” with one virgin female and one male from the Utsunomiya strain, and (2) “hybrid families,” with one virgin female from one of the two strain and a male from the other. We performed all crosses with the same protocol. We isolated parasitized mealybugs in tubes and monitored the emergence of adult *A. burrelli* every morning. We immediately isolated all emerging wasps in separate tubes. We recorded the date and time of emergence and determined the sex of each individual under a microscope. Only females emerging more than 15 min before or after a male were considered to be virgin. Then, we selected individuals for crosses according to the design described above. We placed each couple in a tube with honey for 24 hr for mating. We then released the couple into a plastic box (diameter = 10 cm) containing honey for food and a sprouted potato infested with 10 third‐instar mealybugs, in which we kept them for the rest of their lives.

#### 2.2.2 Inbred versus outbred crosses

At generation G_1_, we mated virgin females by either a brother, in within‐family crosses, or with an unrelated male from another family in between‐family crosses (Figure 1). We used a 2 × 2 factorial design with two types of family (pure vs. hybrid) and two levels of inbreeding (inbred vs. outbred). As well, we used several (up to 11) females and males from each G_0_ mating, so each G_0_ pair was considered as a random block factor. We collected mealybugs that had been parasitized by G_0_ females, and selected emerging adults for mating according to the crossing design for G_1_ adults. Emergence, mating, and the exposure of mealybug hosts to G_1_ adults were as described above.

Twenty days after G_1_ oviposition, we counted the parasitized mealybugs from each box and transferred them to a tube for monitoring of G_2_ emergence. We recorded the number of emerging wasps, and isolated five females from each tube in separate tubes for the estimation of phenotypic traits. We selected one of these females for the measurement of G_2_ realized fecundity by mating with a male randomly selected from another family (this approach made it possible to treat inbred and outbred females similarly). We carried out mating and host exposure as for the G_0_ and G_1_ generations. We kept all the offspring of G_2_ females reaching adulthood in 96% ethanol at 4°C for counting and sex determination.

##### Phenotypic traits

We measured eight life‐history and morphological traits on second‐generation parasitoids (G_2_; Figure 1): we measured development time on males and females, whereas we measured abdomen size, longevity, reproductive success, parasitism rate, success of parasitism, and realized fecundity only for females. We also determined the sex ratio for the G_2_ progeny.

We approximated average development time by the time between the release of the G_1_ parents in the box and the emergence of G_2_ adults. Longevity was the lifetime of one G_2_ female between emergence and death, each female being isolated in a tube with honey and monitored daily. We determined female abdomen size as the mean abdomen size (calculated from three measurements per female) of two females, measured with a binocular microscope coupled to Axiovision^®^ software. Success of parasitism was a binary variable (1 when the female generated at least one parasite mealybug, 0 otherwise). Parasitism rate was the number of mealybugs, of the 10 supplied to a parasitoid female for 48 hr, transformed into a mummy. Reproductive success was a binary variable, with a value of 1 corresponding to the emergence of adults from the parasitized host generated by a female and 0 otherwise. Female realized fecundity was the number of adult offspring emerging from the mealybugs parasitized by a female producing at least one offspring. Since only females can parasitize the host and lay eggs, we considered a high proportion of female as an advantage for biocontrol production and control of the pest. Thus, offspring sex ratio was expressed as the proportion of females among the offspring.

##### Inbreeding depression coefficients

As a quantitative approach for the effect of inbreeding on fitness, we estimated the inbreeding depression coefficient (IDC) for each phenotypic trait (Basamma et al., 2009) as:

$${\text{IDC}}_{W}=\frac{{\overset{¯}{W}}_{\mathrm{outbred}}-{\overset{¯}{W}}_{\mathrm{inbred}}}{{\overset{¯}{W}}_{\mathrm{outbred}}},$$

where $\overset{¯}{W}$ is the mean value for the phenotypic trait considered. Therefore, the IDC is not different from zero in the absence of inbreeding depression, but increases to one as the relative mean performance of inbred individuals decreases. If relative mean performance of inbred individuals is higher than outbred individuals, IDC will decrease from zero to theoretically minus infinite. Note that for the trait “development time,” we considered lower values as a fitness advantage, and thus we multiplied the IDC value by (−1) for this trait (so that higher values correspond to higher fitness). For each IDC, we estimated confidence intervals by a bootstrap method: For each trait and each family type (pure vs. hybrid), we created 1,000 new datasets by resampling with replacement from the raw data for outbred and inbred crosses. From each new dataset, we recalculated means and IDCs. Thus, we obtained 1,000 IDCs and after sorting, we considered values of the 25th and 975th IDCs as boundaries for the 95% confidence intervals.

##### Fitness

If inbreeding significantly affects traits related to survival or reproduction, it should have an impact on individual fitness. We evaluated the effect of inbreeding on fitness by two different methods, both using the number of females produced per female as a proxy for fitness. We used this proxy because both theoretical models and experimental data suggest that, in species with local mating, females ovipositing alone maximize the number of daughters (Hardy, 1994).

We first analyzed the individual data, using raw data for the numbers of daughters produced per G_2_ female as the dependent variable. We then analyzed the group data (four groups: pure inbred, pure outbred, hybrid inbred, hybrid outbred), estimating the same proxy by combining the mean values of several traits for each group. We calculated a fitness index as follows: [success of parasitism] × [reproductive success] × [mating success] × [realized fecundity] × [proportion of females among offspring] (Moreau, Benrey, & Thiery, 2006). This combination cannot be calculated at the individual level, because several of the traits are binary. We took into account success of parasitism as the ability of females to parasitize the host. Reproduction success represented the ability to bypass the immune defences of the host and then to produce offspring. Mating success was a binary variable with a value of 1 when at least one daughter was present in the offspring, which makes sure that a mating event took place. We considered realized fecundity (number of offspring) as an overall fitness assessment. We considered a high proportion of females in the offspring as an advantage for biological control purposes. For comparisons between groups, we estimated 95% confidence intervals via bootstrap resampling as described above for IDCs.

### 2.3 Statistical analysis

We fitted generalized linear mixed models (GLMM) to assess the effects of family type (pure vs. hybrid) and inbreeding level (“inbred” vs. “outbred”) on the variations of all measured phenotypic traits. Both the family of origin of the mother and the family of origin of the father were implemented as random effects. We used a binomial distribution for the analysis of parasitism, reproductive success, and offspring sex ratio. We used a Poisson distribution to analyze realized fecundity, the number of parasitized hosts, and the total number of daughters produced. We also used a lognormal distribution for female longevity. We used a gamma distribution for the analyses of development time. Finally, we used a normal distribution to analyze female abdomen size. We performed type III tests of fixed effects. We used the glmer and ANOVA functions from packages lme4 and car to perform all analyses (Bates, Maechler, Bolker, & Walker, 2015; Fox & Weisberg, 2011) on R version 3.3.4 (R Core Team, 2016). We assessed the power of the statistical analyses as follows. We created 20,000 new datasets by simulating data within the four groups (inbred Utsunomiya, outbred Utsunomiya, inbred hybrid, and outbred hybrid) for each phenotypic trait. For each simulated dataset, we simulated data within each group by using the same sample size and variance as in the observed data, and draw a mean at random within a range corresponding to a 15% deviation around the mean estimated on observations (we considered this range as biologically realistic). We then calculated the difference between means of inbred and outbred groups (referred to as effect size of “level of inbreeding” factor) as well as between Utsunomiya and hybrid groups (referred to as effect size of “family type” factor). We finally performed GLMM analyses for each generated dataset and calculated the proportion of *p*‐values under .05 in a range of effect size from 0% to 60% (iterations for superior effect sizes were not considered). We performed data simulations with rnorm, rpois, and rbinom functions of package stats in R software (R Core Team, 2016). We performed GLMM analysis with glmer function of package lme4 (Bates et al., 2015).

## 3 Results

### 3.1 Phenotypic traits

For each trait, the number of replicates, mean, and standard error are summarized in Table 1. Generalized linear models revealed no effect of the level of inbreeding (*p *>* *.074), family type (*p *>* *.234), or their interaction (*p *>* *.120) on any of the life‐history traits studied (details in Table 2). Inbred and outbred females had similar development times (34.1 ± 0.2 days), abdomen sizes (0.421 ± 3.7 × 10^−3^ mm), longevity (12.9 ± 0.6 days), parasitism rates (18.6% ± 1.9%), success of parasitism (62.9% ± 4.5%), reproductive success (79.5% ± 4.8%), and realized fecundity (44.8 ± 4.7 offspring), regardless of the type of family (pure or hybrid) from which they originated. The sex ratio of the offspring was strongly biased toward females (20.7% ± 2.8% males) and, like other traits, was unaffected by inbreeding. Variances of original paternal and maternal families as random effects approximate 0 and thus suggest no effects from original pure family Utsunomiya or Tottori on male fitness.

**Table 1:** Mean values (±standard errors) of life‐history and morphometric traits measured in *Allotropa burrelli* from different type of families

Traits	Unit	IP		OP		IH		OH	
		*N*	Mean ± *SE*	*N*	Mean ± *SE*	*N*	Mean ± *SE*	*N*	Mean ± *SE*
Success of parasitism	%	30	76.67 **±** 7.85	27	55.56 **±** 9.75	29	62.07 **±** 9.17	30	56.67 **±** 9.20
Parasitism rate	%	30	22.33 **±** 3.91	27	17.41 **±** 3.89	29	18.62 **±** 3.77	30	16.00 **±** 3.31
Reproductive success	%	23	78.26 **±** 12.60	15	66.67 **±** 8.79	18	83.33 **±** 9.04	17	88.24 **±** 8.05
Female realized fecundity	offspring	18	29.78 **±** 5.10	8	47.00 **±** 8.62	14	47.21 **±** 8.67	15	40.07 **±** 7.68
Offspring sex ratio	% females	18	80.78 **±** 2.99	10	69.18 **±** 11.18	15	82.90.10 **±** 2.02	15	80.61 **±** 6.04
Development time	days	67	33.86 **±** 0.29	58	33.72 **±** 0.36	46	34.33 **±** 0.36	46	34.73 **±** 0.44
Female abdomen size	mm	39	0.427 **±** 6.9 × 10^−3^	33	0.413 **±** 6.0 × 10^−3^	26	0.407 **±** 1.1 × 10^−2^	29	0.435 **±** 5.5 × 10^−3^
Longevity	days	40	13.73 **±** 0.99	27	11.67 **±** 1.11	24	12.08 **±** 1.35	27	13.44 **±** 1.41

*N* is the number of replicates. IP, inbred pure; OP, outbred pure; IH, inbred hybrid; OH, outbred hybrid.

**Table 2:** Results of generalized linear model for (a) success of parasitism (b), parasitism rate, (c) reproductive success, (d) female realized fecundity, (e) offspring sex ratio, (f) development time, (g) female abdomen size, and (h) female longevity

Model	*df* = 1	*F*	*p* (>*F*)
(a) Success of parasitism (binary errors, P VC = 0 [*N*_P_ = 30], M VC = 0.64 [*N*_M_ = 25])			
Family type		0.46	.509
Level of inbreeding		3.69	.074
Family type × level of inbreeding		0.14	.714
(b) Parasitism rate (binomial errors, P VC = 0.19 [*N*_P_ = 30], M VC = 0.40 [*N*_M_ = 25])			
Family type		0.25	.627
Level of inbreeding		2.83	.113
Family type × level of inbreeding		0.00	.951
(c) Reproductive success (binary errors, P VC = 0 [*N*_P_ = 20], M VC = 0.44 [*N*_M_ = 18])			
Family type		1.66	.234
Level of inbreeding		0.08	.790
Family type × level of inbreeding		0.49	.506
(d) Female realized fecundity (Poisson errors, P VC = 0.11 [*N*_P_ = 16], M VC = 0.41 [*N*_M_ = 15])			
Family type		0.06	.822
Level of inbreeding		0.99	.393
Family type × level of inbreeding		1.35	.330
(e) Offspring sex ratio (binomial errors, P VC = 0 [*N*_P_ = 16], M VC = 0 [*N*_M_ = 15])			
Family type		1.60	.957
Level of inbreeding		0.004	.303
Family type × level of inbreeding		2.42	.120
(f) Development time (gamma errors, P VC = 8.3 × 10^−5^ [*N*_P_ = 63], M VC = 3.8 × 10^−3^ [*N*_M_ = 59])			
Family type		0.61	.438
Level of inbreeding		0.44	.510
Family type × level of inbreeding		1.54	.219
(g) Female abdomen size (normal errors, P VC = 1.3 × 10^−3^ [*N*_P_ = 33], M VC = 4.4 × 10^−4^ [*N*_M_ = 28])			
Family type		0.07	.794
Level of inbreeding		0.08	.779
Family type × level of inbreeding		4.69	.053
(h) Female longevity (lognormal error, P VC = 0 [*N*_P_ = 30], M VC = 0.21 [*N*_M_ = 28])			
Family type		0.20	.661
Level of inbreeding		0.00	.985
Family type × level of inbreeding		1.30	.272

Details are given in parentheses: error distribution, variance components (VC) for the random effects selected (M, maternal G_0_ family; P,paternal G_0_ family), number of levels for random effects (*N*_M_, number of maternal families; *N*_P_, number of paternal families).

**Figure 2:**
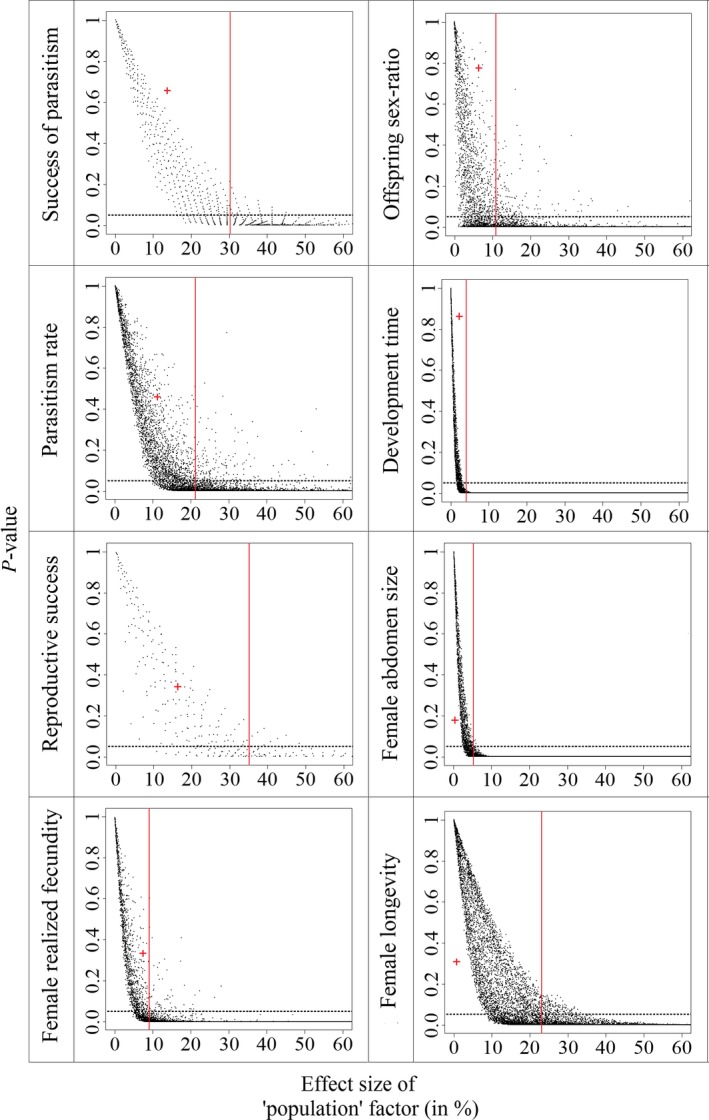
Distribution of *p*‐values depending on effect size (in %) of “family type” factor obtained from simulations for all phenotypic traits. The cross in red shows the obtained data from the experiments. The red line marks the minimum effect size for which the 80% statistical power threshold is obtained. Dashed horizontal line shows the .05 threshold for *p*‐value significance

**Figure 3:**
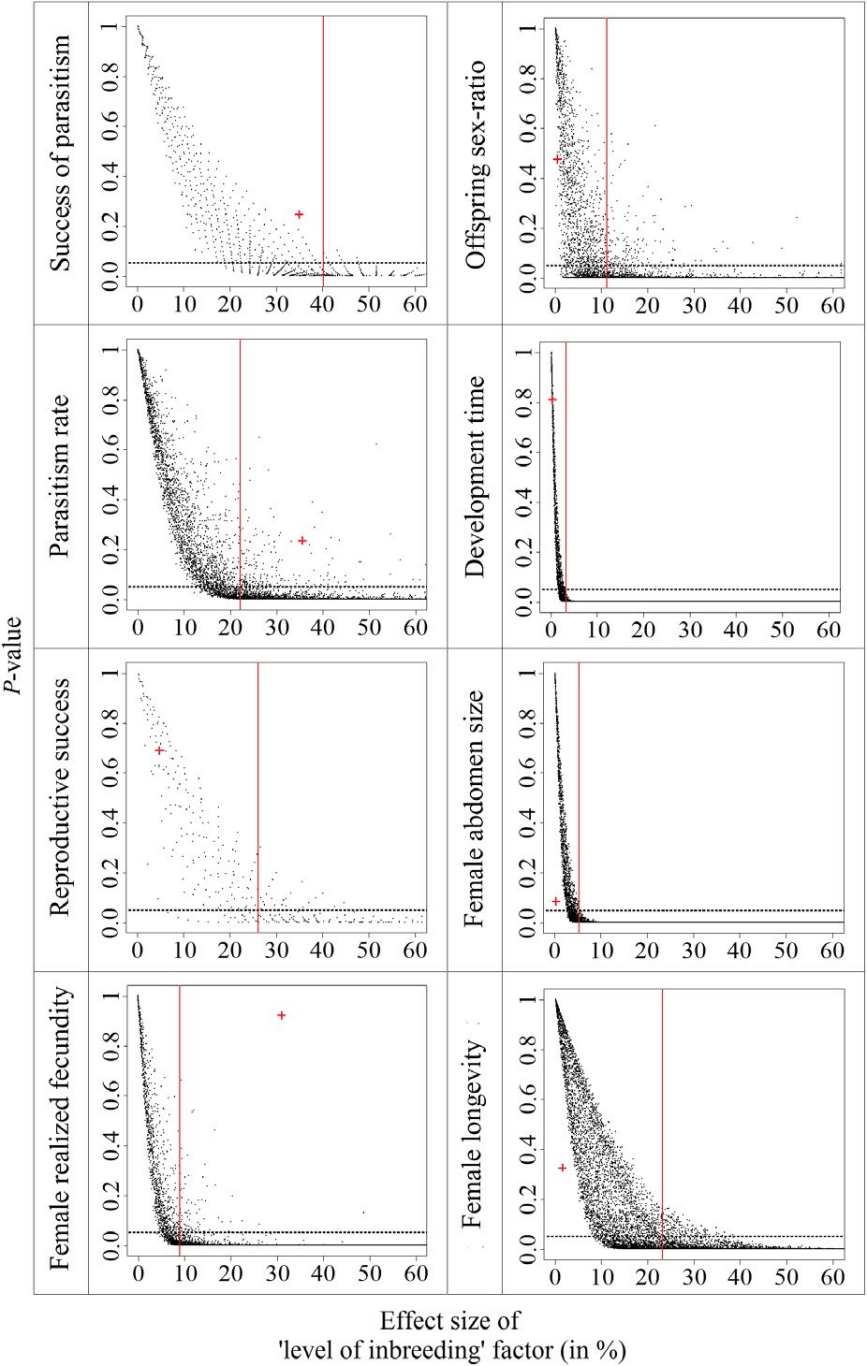
Distribution of *p*‐values depending on effect size (in %) of “level of inbreeding” factor obtained from simulations for all phenotypic traits. The cross in red shows the obtained data from the experiments. The red line marks the minimum effect size for which the 80% statistical power threshold is obtained. Dashed horizontal line shows the .05 threshold for *p*‐value significance

Overall analysis of phenotypic traits thus does not reveal evidence of inbreeding depression in *A. burrelli*. Analyses of statistical power revealed that, for “family type” factor, 80% power threshold is reached for 30%, 21%, 35%, 9%, 11%, 4%, 5%, and 23% effect sizes for success of parasitism, parasitism rate, reproductive success, female realized fecundity, offspring sex ratio, development time, female abdomen size, and female longevity, respectively (Figure 2). For “level of inbreeding” factor, 80% power threshold is reached for 30%, 21%, 35%, 9%, 11%, 4%, 5%, and 23% effect sizes (Figure 3).

#### 3.1.1 Inbreeding depression coefficients

**Figure 4:**
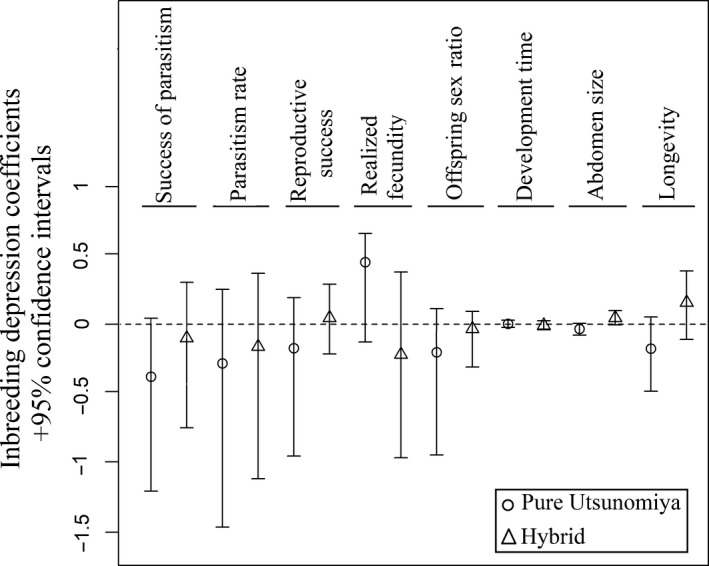
Inbreeding depression coefficients (IDC) and 95% confidence intervals for the eight fitness traits measured in G_2_ individuals

Inbreeding depression coefficients and their 95% confidence intervals were estimated for eight traits, for both pure and hybrid families. For pure families, the IDCs for the eight traits had a confidence interval including zero and were considered to be nonsignificant (Figure 4). For hybrid families, abdomen size had a small but significant IDC (0.07 [95% CI = 0.02–0.12]), whereas the coefficients of the other traits were not significantly different from zero. IDCs thus revealed an absence of inbreeding depression for most fitness traits, the only exception being abdomen size in hybrid families. However, the inbreeding depression for abdomen size was extremely small.

#### 3.1.2 Fitness

**Figure 5:**
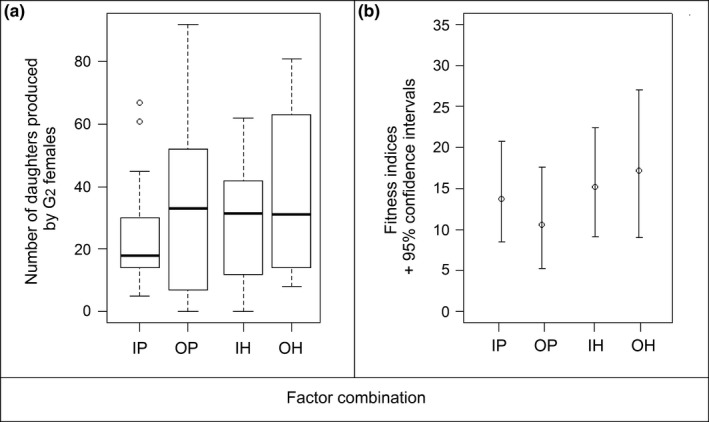
(a) Number of females produced per G_2_ female for each type of family and (b) fitness indices and 95% confidence intervals calculated for each type of family. IP, inbred pure; OP, outbred pure; IH, inbred hybrid; OH, outbred hybrid

The number of daughters produced per female was not affected by the level of inbreeding (*p*‐value* *= .644), in either pure or hybrid families (interaction inbreeding × family type: *p*‐value* *= .145; main effect of family type: *p*‐value* *= .266). On average, G_2_ females produced 31.3 ± 3.06 daughters in 2 days (Figure 5a). These results are consistent with the fitness index estimated from a combination of traits. The estimates and 95% CI obtained suggested that fitness was not dependent upon inbreeding status (Figure 5b).

## 4 Discussion

We evaluated the effect of inbreeding on fitness traits in the parasitoid wasp *A. burrelli*. Specifically, we assessed possible inbreeding depression by comparing phenotypic traits between inbred and outbred individuals. Since we hypothesized that detrimental alleles might have been both purged and fixed in the genetic pool, we established an experimental design to restore heterozygosity to test these two assumptions. Therefore, we carried out the analysis for both pure and hybrid families.

Overall, the obtained results provide no evidence for inbreeding depression in *A. burrelli*. When traits were analyzed independently, with generalized linear models, we found no effect of inbreeding in either pure or hybrid families. The power analysis we performed reveals that the experiment could have led to the detection of significant effects of the “level of inbreeding” on parasitism rate and female realized fecundity, even with effect sizes lower than those observed (35.5% and 31.0%, respectively). Hence, the absence of significant effect of “level of inbreeding” on these two traits in the GLMM analysis is likely caused by random variations, rather than an insufficient statistical power. Moreover, although our statistical power is rather low for some other phenotypic traits, the very small effect size observed suggests no occurrence of a biologically relevant pattern at these traits. However, the “level of inbreeding” effect size observed for parasitism success (34.8%) is slightly lower than the threshold at which statistical power reaches 80% (Figure 5), and yet could correspond to a biologically important difference between inbred and outbred individuals. However, at this trait, even if the 34.8% effect size was found significant in the GLMM, it would correspond to a higher trait value in inbred individuals. Hence, this result would not challenge the main conclusion that no major evidence for inbreeding depression was detected. In addition, the same trend was observed for IDCs for which seven out of the eight IDCs calculated (the exception being abdomen size in hybrid families) show no evidence for inbreeding depression in either pure Utsunomiya or hybrid families, because the confidence intervals included zero. Moreover, overall fitness evaluation through the calculation of fitness indices or the assessment of daughter production showed no effect of family type or inbreeding level. The obtained results agree with published findings concerning the effects of inbreeding in haplodiploids (Charlesworth & Willis, 2009; Henter, 2003; Werren, 1993). The genetic load of *A. burrelli* had probably been purged through haploid males on one hand and by the systematic inbreeding resulting from its gregarious behavior on the other.

One key finding of this work was that the mass‐reared populations of *A. burrelli* used for the biological control program in France are unlikely to be strongly affected by inbreeding, at least in laboratory conditions. As a consequence, no specific measures to prevent inbreeding are required in mass‐rearing protocols, other than trying to ensure that the population size remains large, to avoid losses of genetic diversity, which could lead to a decrease in adaptability (García‐Dorado, 2015). The present findings suggest the possibility to create inbred lines of *A. burrelli*. Therefore, we generated and maintained highly inbred lines by crossing brothers and sisters over several generations, to capture unique genotypes that may be needed to reintroduce into the population in the future if there is a loss of genetic diversity from the main mass‐reared populations. This method has already proved effective in *Chiasmia assimilis* Warren (Lepidoptera: Geometridae), in which genetic diversity is similar in conventionally reared insects and in accumulated isofemale lines (Wardill et al., 2004).

## Conflict of Interest

None declared.
